# The “Magnesium Sacrifice” Strategy Enables PMMA Bone Cement Partial Biodegradability and Osseointegration Potential

**DOI:** 10.3390/ijms19061746

**Published:** 2018-06-12

**Authors:** Qingpan Zhai, Fengxuan Han, Zhiwei He, Chen Shi, Pinghui Zhou, Caihong Zhu, Qianping Guo, Xuesong Zhu, Huilin Yang, Bin Li

**Affiliations:** 1 College of Chemistry, Chemical Engineering and Materials Science, Orthopaedic Institute, Soochow University, Suzhou 215000, China; zhaiqingpan@126.com (Q.Z.); fxhan@suda.edu.cn (F.H.); m13913980758@163.com (Z.H.); zphdoctor@126.com (P.Z.); zhucaihong@suda.edu.cn (C.Z.); guoqianping@suda.edu.cn (Q.G.); zhuxs@126.com (X.Z.); suzhouspine@163.com (H.Y.); 2 Department of Biomedical Engineering, National University of Singapore, 117583 Singapore, Singapore; shichen.ce@gmail.com; 3 China Orthopaedic Regenerative Medicine Group (CORMed), Hangzhou 310000, China

**Keywords:** bone cement, PMMA, magnesium, partial degradation, osseointegration

## Abstract

Poly (methyl methacrylate) (PMMA)-based bone cements are the most commonly used injectable orthopedic materials due to their excellent injectability and mechanical properties. However, their poor biocompatibility and excessive stiffness may cause complications such as aseptic implant loosening and stress shielding. In this study, we aimed to develop a new type of partially biodegradable composite bone cement by incorporating magnesium (Mg) microspheres, known as “Mg sacrifices” (MgSs), in the PMMA matrix. Being sensitive to the physiological environment, the MgSs in PMMA could gradually degrade to produce bioactive Mg ions and, meanwhile, result in an interconnected macroporous structure within the cement matrix. The mechanical properties, solidification, and biocompatibility, both in vitro and in vivo, of PMMA–Mg bone cement were characterized. Interestingly, the incorporation of Mg microspheres did not markedly affect the mechanical strength of bone cement. However, the maximum temperature upon setting of bone cement decreased. This partially biodegradable composite bone cement showed good biocompatibility in vitro. In the in vivo study, considerable bony ingrowth occurred in the pores upon MgS degradation. Together, the findings from this study indicate that such partially biodegradable PMMA–Mg composite may be ideal bone cement for minimally invasive orthopedic surgeries such as vertebroplasty and kyphoplasty.

## 1. Introduction

Poly(methyl methacrylate) (PMMA)-based bone cements have been used as a grouting agent in orthopedic surgeries for more than 50 years [1]. Each year, PMMA bone cements are used in more than 75,000 vertebroplasty and kyphoplasty surgeries and more than 700,000 knee and hip replacement surgeries in the Medicare population in the United States [2,3]. Recently, several new types of bone cement such as calcium phosphate cement and calcium sulfate cement have been developed as a substitution for PMMA [4,5]. Nevertheless, PMMA still remains the most widely used bone cement material in total joint arthroplasty, vertebroplasty, and kyphoplasty due to the decent injectability and mechanical properties [6,7]. However, further clinical applications of PMMA as bone cement are impeded by several limitations. For example, a large number of cases have confirmed the loosening and shifting of implanted PMMA bone cement as one of the major causes of surgical failure [8]. The interface between bone and PMMA cement may be unstable due to the hydrophobicity and biologically inactivity of PMMA. Moreover, the relatively high elastic modulus of PMMA bone cement may also result in stress shielding and induce secondary fractures of the adjacent vertebral bodies [9].

To date, a number of strategies have been employed to overcome these shortcomings by improving the interface and interaction between bone and PMMA-based bone cements. Compositing non-bioactive PMMA cements with other bioactive fillers represents an effective approach. For example, Kim et al. prepared bioactive bone cement (BBC) using natural bone powder (hydroxyapatite) and chitosan powder, which showed better biocompatible and osteoconductive properties than pure PMMA in vivo [10]. Bone cement containing titania (TiO_2_) and silanized TiO_2_ particles prepared by Goto et al. was reported to show significantly increased osteoconductivity [11,12]. Jiang et al. and Li et al. incorporated mineralized collagen (MC) into PMMA bone cement, resulting in a MC-PMMA cement with high mechanical properties, good biosafety and excellent biocompatibility [13,14]. In another study, Sa et al. evaluated the effects of incorporation of calcium phosphate (CaP) particles on the physicochemical properties and mineralization capacity of the PMMA cements in vitro [15]. Improved mineralization capacity of PMMA cement was achieved without compromising the mechanical properties. However, some problems still remain, including displacement of bone cement in body and insufficient module of porous bone cement after degradation [16]. Besides the physicochemical properties, the microstructures of bone cement also dramatically impact the bone regeneration after implantation. Biomaterials with interconnected porous structures have been shown to promote bone formation better [17]. However, the current strategy of mixing bone cement and bioactive filler is not able to change the microstructures of bone cement.

As a kind of biodegradable and biocompatible metal that has similar mechanical properties to bone, Mg has recently attracted increasing attention for orthopedic applications [18,19]. In a physiological environment, Mg gradually degrades and results in high mineral apposition rates and increased bone mass around the implant. As an essential element for bone growth, Mg can promote the deposition of calcium phosphate [18]. Yang et al. found that during the degradation of Mg implants in diabetic rats, the serum content of Mg ions and bone mineral content of Ca and P increased significantly, suggesting that Mg might play a role in the process of bone healing in patients with osteoporosis through promoting new bone formation [20]. Other studies also show that Mg can enhance the expression of some growth factors, such as bone morphogenetic protein-2 (BMP-2) and vascular endothelial growth factor (VEGF), which could promote bone formation and be beneficial for reducing the harmful effect of osteoporosis [21,22]. Moreover, previous in vivo and in vitro studies have shown that the surrounding tissue has no systematic inflammatory reaction or affection after Mg implanted [23]. Hence, Mg may be used as a potential porogen in bone cement.

**Figure 1:**
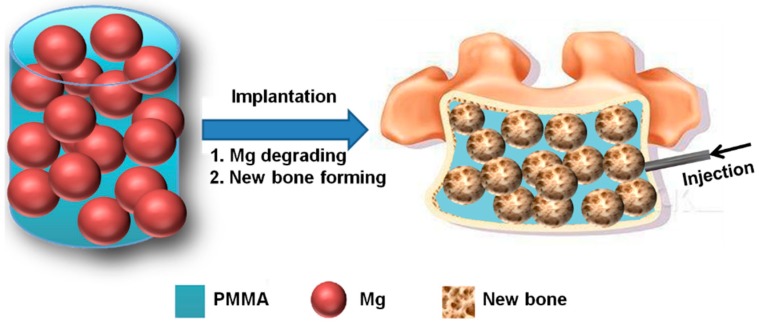
Schematic illustration of a partially degradable PMMA bone cement based on the “magnesium sacrifice” strategy.

The objective of this study, therefore, was to prepare a new type of partially degradable cement with bioactivity and interconnective macroporous structure using an “Mg sacrifice” strategy. Here, sacrifice of Mg refers to the degradation and subsequent release of Mg ions from the Mg microspheres-embedded PMMA matrix under physiological microenvironment. As a result, the Mg ions and interconnective pores will promote bony ingrowth into the PMMA matrix (Figure 1). The performance, including mechanical properties, curing characteristics, in vitro and in vivo biocompatibility, of PMMA bone cement incorporated with Mg microspheres were evaluated.

## 2. Results

### 2.1. Preparation and Characterizations of PMMA–Mg Bone Cements

**Figure 2:**
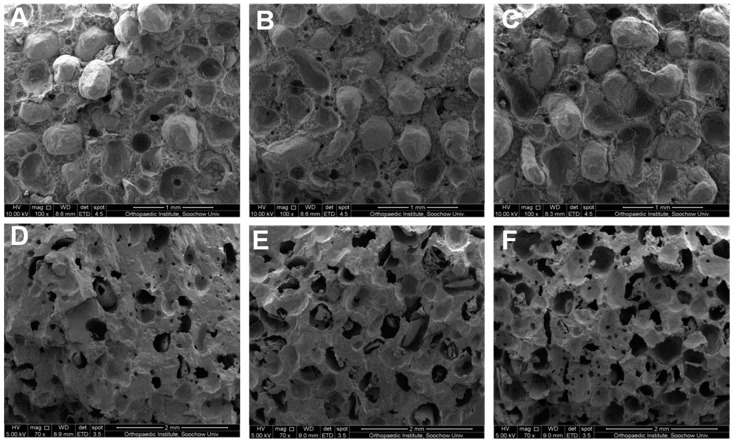
SEM images of composite bone cements. (**A**,**D**) PMMA–25Mg; (**B**,**E**) PMMA–41Mg and (**C**,**F**) PMMA–58Mg bone cements before (**A**–**C**) and after (**D**–**F**) degradation. Scale bars, 1 mm (**A**–**C**) and 2 mm (**D**–**F**).

The cross-section morphology of PMMA–Mg bone cements before and after degradation is shown in Figure 2. The Mg microspheres are randomly distributed in the PMMA matrix. Part of Mg microspheres can contact with each other, the contact ratio increase with Mg microspheres contents. After degradation, porous PMMA matrix could be seen. The pore sizes of PMMA matrix are in the range of 300–500 μm. Furthermore, the porosity and interconnectivity of the bone cement also increased with the Mg microspheres content. Apparently, the addition of Mg microspheres dramatically affected the internal microstructure of bone cement. Mg microspheres can be good porogen to achieve porous PMMA bone cements. When a suitable density of Mg microspheres was achieved, interconnected pores formed, which might favor nutrient delivery and waste discharge.

**Figure 3:**
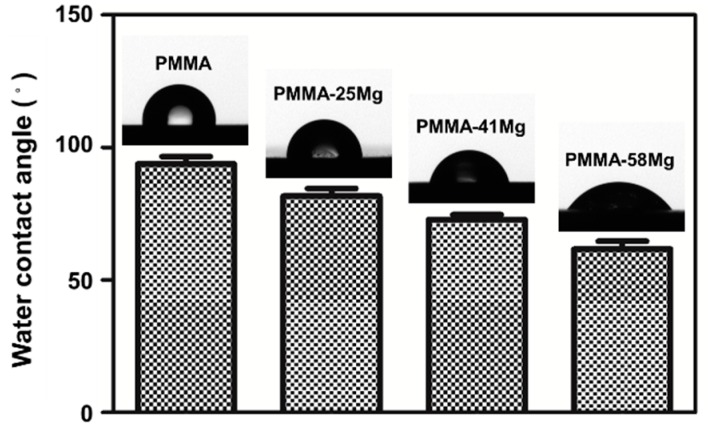
Water contact angle of PMMA, PMMA–25Mg, PMMA–41Mg, and PMMA–58Mg.

Figure 3 shows typical water contact angles of PMMA cement and PMMA–Mg composite cements. PMMA is a hydrophobic material with a water contact angle of 95.2° ± 5°. The water contact angle decreases with incorporating Mg microspheres. The water contact angle (average value) was reduced to 81.7°, 72.8°, and 61.7° for PMMA–25Mg, PMMA–41Mg, and PMMA–58Mg composite cements, respectively.

**Figure 4:**
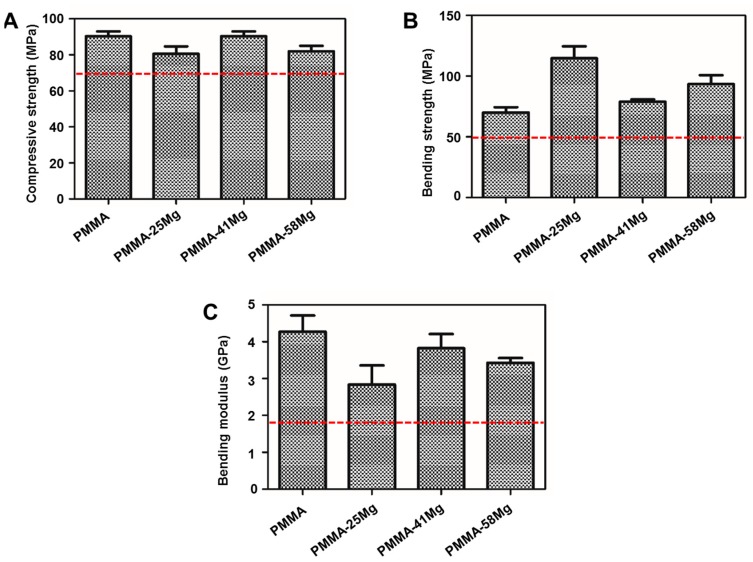
The mechanical properties of PMMA–Mg composite bone cement. (**A**) Compressive strength; (**B**) bending strength and (**C**) bending modulus. Red dashed line shows the ISO 5833 industry standard.

The mechanical properties of PMMA bone cement, PMMA–25Mg, PMMA–41Mg, and PMMA–58Mg composite cements are shown in Figure 4. The compressive strength does not show significant differences between groups (Figure 4A). PMMA–25Mg shows a minimal compressive strength of 80.30 ± 3.26MPa, which is still higher than the industry standard (no less than 70 MPa according to ISO 5833; red dashed line in Figure 4). The bending strength increases but bonding modulus decreases with the addition of Mg microspheres (Figure 4B,C). PMMA–41Mg, exhibiting the best mechanical properties, will be used in the following experiments.

**Figure 5:**
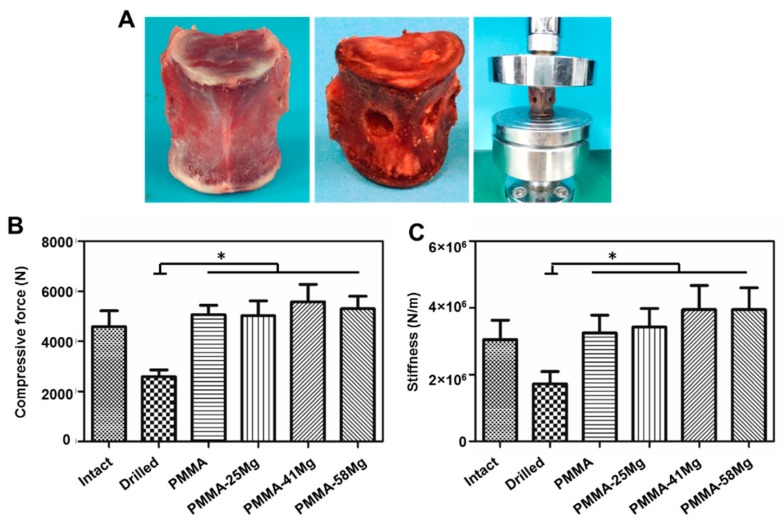
Vertebral body defect augmentation experiments with bone cement in vitro. (**A**) The gross morphology of vertebrae and drilled vertebrae and descriptive graph of the compressive test; (**B**) compressive strength and (**C**) stiffness of intact, drilled PMMA and PMMA–Mg cements filled vertebrae. * *p* < 0.05.

The mechanical properties of drilled vertebrae filled with different bone cements are shown in Figure 5. The gross morphology of vertebrae and drilled vertebrae and descriptive graph of the compressive test can be seen in Figure 5A. Both the compressive strength and stiffness of drilled vertebrae decrease (Figure 5B,C). However, the filling of PMMA bone cement, PMMA–25Mg, PMMA–41Mg, and PMMA–58Mg composite cements in drilled vertebrae markedly increased their mechanical strength. Moreover, the vertebrae filled with both PMMA and PMMA–Mg bone cements had similar stiffness and strength as native vertebrae.

### 2.2. Curing Characteristics

**Figure 6:**
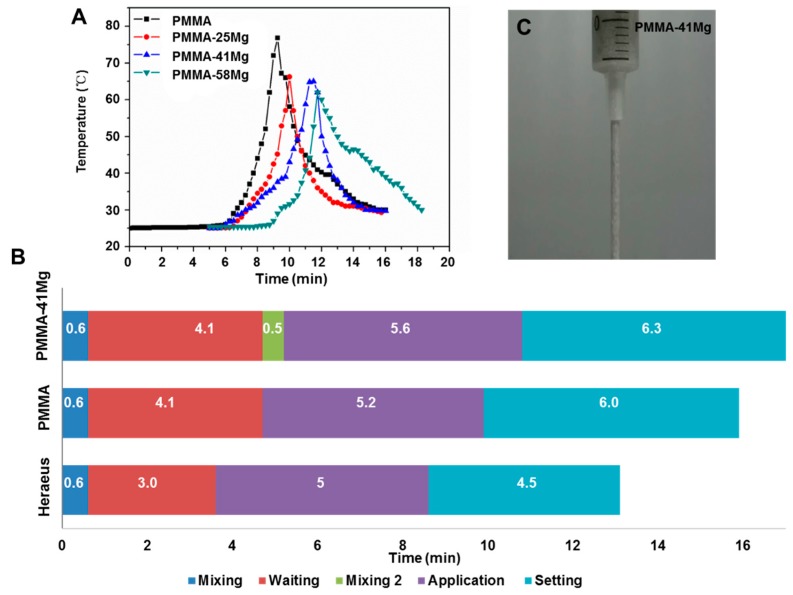
(**A**) Heat evolution curves for the setting reactions of bone cement; (**B**) solidification characteristics of PMMA–41Mg measured at 25 °C, with PMMA bone cement and Heraeus cement as control; (**C**) the injectability of PMMA–41Mg bone cement.

Figure 6A shows the typical exotherms of polymerization of the composite bone cements. The peak temperatures of PMMA, PMMA–25Mg, PMMA–41Mg, and PMMA–58Mg are 76.8, 66.24, 64.8 and 52.7 °C, respectively. The peak temperatures of PMMA bone cement polymerization obviously decreased after the addition of Mg microspheres. The characteristic solidification processes of PMMA–41Mg bone cements at 25 °C are shown in Figure 6B. The mixing period, waiting period, application period, and setting period of PMMA–41Mg were 0.6, 4.1, 5.6 and 6.3 min, respectively. Elongated setting time was observed for PMMA–41Mg bone cement compared with PMMA bone cement. After addition of Mg beads in PMMA bone cement, the PMMA–41Mg still showed good injectability (Figure 6C). This composite bone cement could be easily squeezed out from syringe, permitting its potential use in clinical treatments.

### 2.3. In Vitro Degradation Tests

**Figure 7:**
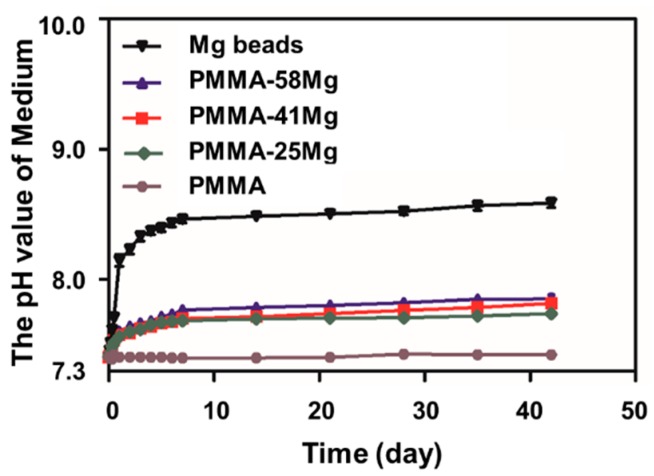
The changes of pH value of SBF medium containing Mg beads, PMMA, PMMA–25Mg, PMMA–41Mg, and PMMA–58Mg bone cements.

Degradation of Mg results in the increase of pH of medium and release of H_2_ [24]. The degradation characteristics of Mg microspheres, PMMA, PMMA–25Mg, PMMA–41Mg, and PMMA–58Mg bone cements are shown in Figure 7. The pH value of pure PMMA bone cement almost did not change at all. Simulated body fluid (SBF) medium containing pure Mg microspheres increased from 7.4 to 8.5 after seven days. The pH value tended to stabilize after seven days. The pH increment of PMMA–Mg groups was lower than pure Mg microspheres, with the pH remaining below 8 even after 42 days. In addition, the final pH of PMMA–25Mg was lower than that of PMMA–41Mg and PMMA–58Mg. These results indicate that the Mg microspheres within PMMA matrix were able to slowly degrade to form pores in situ. The obvious changes of pH value were seen in PMMA–Mg groups, though there was no significant difference between the three groups. However, the peak values for these samples were all less than 8.0. On the other hand, the PMMA matrix delayed the degradation of Mg.

### 2.4. Cell Culture

**Figure 8:**
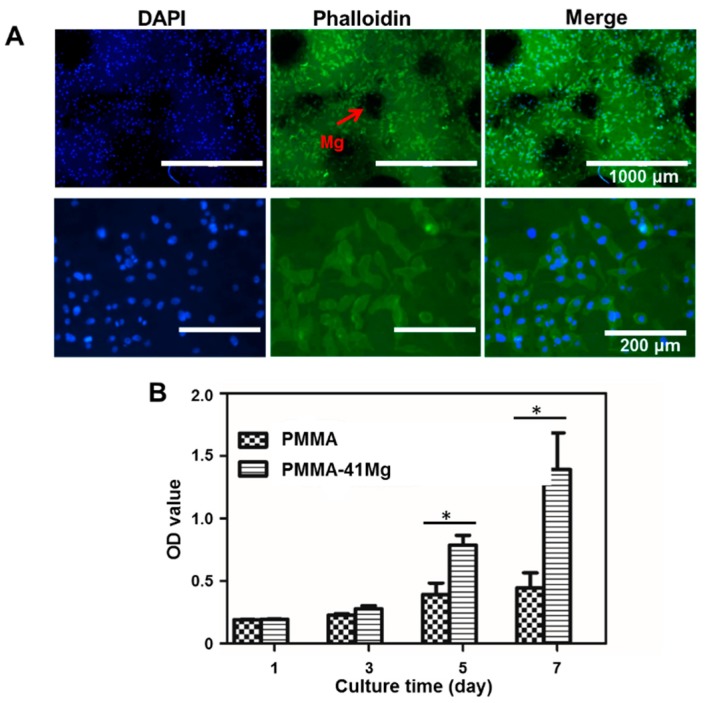
(**A**) Fluorescent staining images of MG-63 cell adhesion on PMMA–41Mg bone cements and (**B**) proliferation of MG-63 cell on PMMA–Mg bone cement. * *p* < 0.05.

The culture of MG-63 cell on PMMA–Mg bone cements is shown in Figure 8. To evaluate whether the cells could attach on the surface of bone cements, the cell attachment experiment was carried out. Clearly, MG-63 cells firmly attached onto the surface of PMMA–41Mg cements with good morphology (Figure 8A). The circular black areas in fluorescent staining images are Mg microspheres inside the cements, because Mg could not be stained by 4′,6-diamidino-2-phenylindole (DAPI) or phalloidin. Further, CCK-8 tests at one, three, five, and seven days of culture were carried out in order to assess the cell proliferation in the bone cements. Apparently, the OD value of all samples increased with culture time. Moreover, the OD values for PMMA–41Mg group were consistently higher than those of control group (PMMA group) along the entire course of culture (Figure 8B). Therefore, it is clear that the bone cements in this study all supported cell attachment and proliferation. Moreover, PMMA–41Mg composite bone cement showed better biocompatibility compared to PMMA bone cement.

### 2.5. Animal Tests of PMMA–Mg Cements

**Figure 9:**
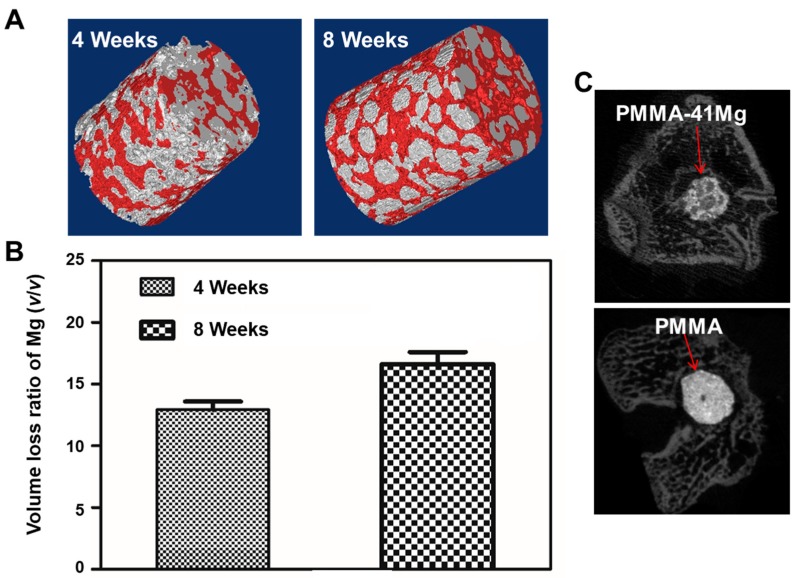
(**A**) Micro-CT reconstructed images of implanting during the four weeks and eight weeks (red indicates PMMA and gray indicates Mg microspheres); (**B**) volume loss ratio of Mg and (**C**) micro-CT images of PMMA–41Mg and PMMA after implantation for eight weeks.

**Figure 10:**
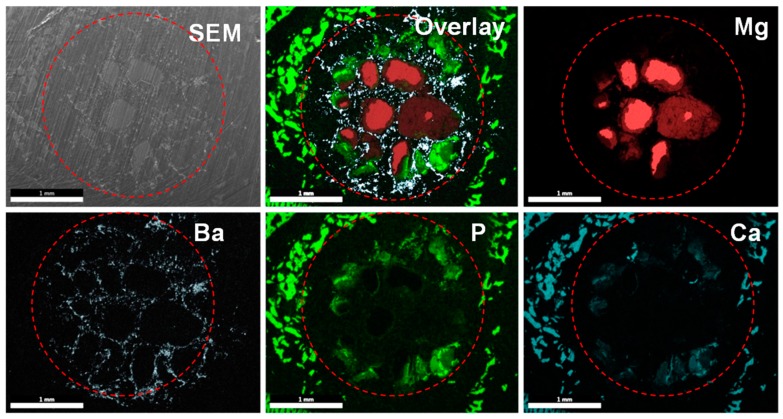
SEM and element mapping of Mg, Ba, P, and Ca of PMMA–41Mg and after implantation in rats for eight weeks. The red circles show roughly the boundary of bone cement. Scale bars, 1 mm.

To assess the in vivo degradation property of PMMA–Mg, bone cements samples were implanted into bilateral pockets of dorsal muscle. The micro-CT reconstructed images show the degradation performance of implanted PMMA–41Mg after four weeks and eight weeks in muscle (Figure 9A). Loss volumes ratio of Mg microspheres in above cement is shown in Figure 9B. At four weeks, the volume loss ratio (average value) of Mg is 12.9%. The degradation quantity of Mg microspheres in PMMA increases with implantation time. After implantation into bilateral pockets of dorsal muscle for eight weeks, the volume loss ratio (average value) of Mg reaches to 16.6%. Micro-CT images showed in Figure 9C are PMMA–41Mg and PMMA bone cement after implantation for four and eight weeks. It is obviously that PMMA bone cement has an apparent detachment with surrounded bone. However, there exists some visible callus around PMMA–41Mg composite bone cements. Better bone integration is obtained using PMMA–41Mg composite bone cements. Moreover, the PMMA–41Mg composite bone cements show some black areas corresponding to existing Mg microspheres. Element mapping of PMMA–Mg bone cement harvested at eight weeks, obtained by SEM and energy dispersive X-ray spectroscopy (EDX), is shown in Figure 10. After eight weeks being implanted, Ca element and P element are assembled in PMMA–Mg bone cement surrounding, which may indicate regenerated bone. Furthermore, Ca element and P element were also seen in the center of bone cements, especially the edge of Mg microspheres. The in vivo experiment results show that the Mg microspheres in PMMA–41Mg were able to slowly degrade in vivo and resulted in pores for bone ingrowth, which favored the integration between the bone tissue and bone cement.

## 3. Discussion

In this study, we developed a new type of partially degradable PMMA bone cement using a “Mg sacrifice” strategy, in which degradable Mg microspheres were incorporated into conventional PMMA bone cement. This composite bone cement resulted in the formation of interconnected pores, which favored bony ingrowth, after degradation of Mg microspheres in a physiological environment (Figure 2) [25,26].

The incorporation of Mg microspheres also affected the solidification process of bone cement. As the content of Mg microspheres increased, the liquidity of cement decreased. While PMMA–25Mg and PMMA–41Mg bone cement still had good injectability, PMMA–58Mg bone cement was almost non-injectable. The maximum temperature decreased and the application time and setting time increased as the content of Mg microspheres increased (Figure 6). Since Mg has high thermal conductivity, the heat produced from polymerization could be absorbed and conducted quickly by Mg microspheres. Therefore, the maximum temperature during bone cement formation decreased. Similar findings have been reported in previous works by other authors [12,27]. The excessive polymerization temperature of PMMA bone cement may result in thermal necrosis of surrounding tissue [28,29,30]. According to ISO 5833-2002, the bone cement should have an application time of 5 ± 1.5 min and a setting time of 3–15 min. Our PMMA–Mg bone cements could provide relatively longer setting time and lower polymerization temperature, both of which favored its potential clinic applications.

Mechanical strength is one of the most important properties of bone cements; inadequate strength means they cannot be used clinically. For example, although calcium phosphate cement and calcium sulfate cement have good biocompatibility, the weak mechanical properties limit their application in a clinical setting. In previous studies, degradable carboxymethylcellulose, alginate, and gelatin microparticles were used as porogens in PMMA bone cement, but these degradable composite bone cements showed low mechanical strength [31]. To improve the mechanical properties of PMMA composite bone cement, magnesium was used as a porogen in this study due to its advantage of high mechanical strength close to cortical bone. When bone cements were compressed and bent, the Mg microspheres could absorb most of the stress, so the mechanical strength of PMMA–25Mg and PMMA–41Mg bone cements was better than that of PMMA cement. However, when the content of Mg microspheres exceeded 58%, the PMMA matrix, which was the major component to bear the external mechanical forces, became too thin—not strong enough to withstand the impact of external mechanical forces. As a result, the mechanical properties of bone cements decreased when excessive Mg microspheres were incorporated. The vertebral body defect augmentation experiment in this study proves that the strength of PMMA–Mg composite bone cement can meet the needs of clinical practice (Figure 5B,C). However, the degradation rate of pure Mg is very fast in physiological environments, which hampers its clinical applications. In this study, PMMA matrix acted as a physical barrier to slow down the degradation of Mg. With the degradation of Mg, the pH value of surrounding body fluid increases and the mass of Mg decreases. PMMA–Mg showed significantly lower degradation rate than pure Mg microspheres according to the measurement of pH change of body fluid (Figure 7). It is noted that not all the added Mg microspheres in PMMA–Mg composite bone cement degraded even after a long period of time. This is because some Mg microspheres were fully wrapped by PMMA, which prevented their contact with body fluid. It has been reported that a fast increase of pH in a local area severely disrupts cell proliferation, differentiation, and viability on the surface of implants and consequently induces chronic tissue inflammatory reactions and blood clots [32,33]. Body fluids in human are a buffer system; slow Mg degradation does not significantly alter the pH value near the host tissue. Our animal experiments indicate that there is no abnormality in the tissues surrounding the Mg-containing implant. It has been shown by numerous previous studies that the microstructures of implanted biomaterials with high porosity and large pore sizes can facilitate bone ingrowth and osseointegration. For example, He et al. demonstrated that porous bone cement allowed bone ingrowth and enhanced bone bonding [31]. Relatively larger pores (>300 μm) are better for osteogenesis than small pores, since they allow vascularization and better oxygenation [17]. In our study, biodegradable Mg microspheres were incorporated in PMMA bone cement to construct macroporous and interconnected microstructure in the cement. Importantly, different pore sizes and interconnectivity could be readily achieved by changing the Mg microsphere content in bone cement. 

Both in vitro and in vivo studies demonstrated that PMMA–Mg composite bone cements are biocompatible and osteoconductive. Under ideal conditions, the degradation time of Mg microspheres should match the time of new bone growth, because the mechanical properties of the porous PMMA may not be strong enough to support the adjacent bone tissue [34]. Therefore, the bioactivity of Mg is an important factor to ensure the implant site is stable. The cell morphology on cement indicates that PMMA–Mg bone cements were able to provide a favorable surface for cell adhesion and growth, which echoes the findings from a previous study by Kim et al. [35]. In vitro experiments in this study have also demonstrated that PMMA–Mg bone cements could well support the adhesion and growth of MG-63 cells (Figure 8). It was also discovered in a previous study that cells appeared to prefer a rough and hydrophilic surface rather than a smooth and hydrophobic surface [36]. The change from hydrophobic surface to hydrophilic surface by adding Mg microspheres to PMMA bone cement may also be helpful for cell adhesion and growth (Figure 3). A previous study also showed that PMMA-based porous cement could promote osseointegration [37,38]. New bone could grow into the pores of degraded Mg microspheres within the PMMA–Mg bone cement, which would increase the interface bonding strength and result in the long-term stability of the cement. A lot of previous studies have confirmed that Mg is a good choice as an orthopedic implant material because it can promote the deposition of calcium phosphate and new bone formation [18,20,21,22]. From micro-CT images and EDX maps (Figure 9 and Figure 10), it is clear that Mg-containing PMMA bone cement could promote mineralization and bone tissue generation. There was space between PMMA bone cement and the surrounding bone tissues. However, newly formed bone tissue could be observed at the interface between bone and PMMA–41Mg cement. These results prove that the addition of Mg beads in PMMA bone cement indeed promoted new bone formation. The enhanced bone ingrowth and bone formation ability of PMMA–41Mg cement was further proven by element mapping in Figure 10. After eight weeks of implantation, Ca and P elements were seen at the edge and center of bone cements, especially along the edge of Mg microspheres. These results show that new bone formation might have occurred along with the degradation of Mg microspheres. The Mg^2+^ ions and pores produced in the bone cement also favored bone regeneration and ingrowth. Although long-term animal studies and in vitro studies for osteogenic behaviors remain to be performed, our “Mg sacrifice” strategy appears to effectively enable PMMA bone cement partial biodegradability and bone ingrowth potential. Therefore, it may provide a promising approach and material for osteoporotic compression fracture treatments.

## 4. Materials and Methods

Mg microspheres (particle size: 300–500 μm), PMMA prepolymer and *N*,*N*-dimethyl-*p*-toluidine (DMPT) were supplied by Alfa Aesar Chemical Co., Ltd. (Shanghai, China). Benzoyl peroxide (BPO), barium sulfate (BaSO_4_), and methyl methacrylate (MMA) were purchased from Sinopharm Chemical Reagent Co., Ltd. (Shanghai, China). Cell Counting Kit-8 reagent (CCK-8) was purchased from Dojindo (Kumamoto, Japan).

### 4.1. Preparation of Partially Degradable PMMA–Mg Composite Bone Cements

PMMA–Mg composite bone cement consisted of solid, liquid, and degradable Mg microspheres. The solid to liquid ratio was 2:1 for preparing the experimental cements. Solid components contained 88.5 wt % PMMA concentration, 10 wt % BaSO_4_ and 1.5 wt % BPO. Liquid components contained 98.9 wt % MMA and 1.1 wt % DMPT. BPO and DMPT were used as initiator accelerator for polymerization, respectively. BaSO_4_ was used as the X-ray contrast medium. Three compositions of bone cements with various contents of Mg microspheres were prepared in this study, as shown in Table 1. The weight percentage of Mg was relative to the total weight of bone cement. Mg microspheres were added to PMMA bone cement at 5 min after the mixing of liquid and solid components. Then the composite bone cement was solidified in stainless-steel molds for further tests.

### 4.2. Characterizations of PMMA–Mg Bone Cements

The morphologies of the prepared bone cement were observed through scanning electron microscopy (SEM, Quanta250, FEI, Hillsboro, OR, USA) with an EDX (TEAM EDS, EDAX, Berwyn, PA, USA) detector attached for elemental analysis. Bone cements with a diameter of 6 mm and a height of 12 mm were prepared as in Section 4.1; after solidification, the samples were cut in half, and the section of the sample was observed by SEM. The applied voltage was 10 kV and the fracture surfaces of samples were sputtered with platinum before SEM observation.

The mechanical properties of the bone cements were tested using a mechanical testing machine (E10000, Instron, Boston, MA, USA). In brief, five bone cement specimens with a diameter of 6 mm and a height of 12 mm in each group were tested for compressive strength and five bone cement specimens (length, 70 mm; height, 3.5 mm; width, 10 mm) in each group were tested for bending strength and bonding modulus in accordance with International Standard ISO 5833:2002. 

A KRÜSS DSA25 contact angle equipment (Hamburg, Germany) was utilized to determine the contact angles of the bone cements. Bone cements with a diameter of 12 mm and a height of 3 mm were prepared as in Section 4.1. Water was dropped onto the bone cements for the measurement. The average of the five tests was taken when analyzing the data.

### 4.3. Vertebral Body Augmentation Tests

Fresh sheep (40–50 kg) vertebrae (L2–L6) were used for vertebral body augmentation tests. After the vertebrae were disarticulated and the discs excised, the posterior elements were removed to facilitate mechanical testing. A 6-mm drill hole was made over the lateral cortex of vertebra. A cavity was created in the vertebral body with a spoon curette. On each vertebral body, the recessed surface was filled with bone cement (inclined ≤1°). After 30 min of curing at room temperature, each vertebra was seated in a loading fixture and compressed at a rate of 5 mm/min using a material compression machine (HY-1080) until the anterior height of the vertebra was decreased by 25%. The strength and stiffness of each vertebra were measured. Strength and stiffness were defined as the peak load and the slope of the force versus the deformation curve, respectively [39].

### 4.4. Curing Characteristics

The setting time of the bone cements was determined according to ISO 5833. We divided the solidification into five periods including mixing, waiting, second mixing, application and setting. Commercial PMMA bone cements (Heraeus, Hanau, Germany) were tested as controls. The tests were performed at 25 °C. The mixing time is the period of time from the first addition of liquid to the powder to the point when stirring stopped. The waiting time refers to the period of time from the stopping of stirring until the mixture could be cleanly separated from a gloved finger. The second mixing time refers to the time when Mg microspheres were added into PMMA matrix and mixed for 5 s. The application time refers to the period of time that a surgeon could easily handle the cement and inject it. The setting time was determined as the time corresponding to the average value of the maximum and the ambient temperature. The temperature change during the setting reaction was measured using a thermocouple according to ISO 5833.

### 4.5. In Vitro Degradation of Bone Cement

The in vitro degradation of PMMA–Mg bone cements in SBF was tested by testing the pH value of SBF every day. Five bone cement specimens in each group were immersed in SBF at 37 °C, and pure Mg microspheres were tested as a control group. The total content of Mg components maintained consistency between groups.

### 4.6. Cell Culture

#### 4.6.1. Cell Attachment Experiment

MG-63 cells were seeded on the cements at a concentration of 1 × 10^5^ cells/cm^2^ and allowed to adhere during incubation at 37 °C in 5% CO_2_ for 24 h. Then the specimens were washed with phosphate-buffered saline (PBS) to remove non-adherent cells and fixed in ice-cold 4% paraformaldehyde (PFA) for 40 min. After washing with PBS and permeabilizing of cells with 20% methanol for 20 min, the cells were stained with dihydrochloride (DAPI) and phalloidin. Imaging was conducted using a fluorescent inverted microscope (Zeiss Axiovert 200, Carl Zeiss, Oberkochen, Germany).

#### 4.6.2. Cell Proliferation Assay

MG-63 cells were seeded into 96-well cell culture plates at a density of 1 × 10^5^ cells/cm^2^. After culturing at 37 °C in a humidified incubator with 5% CO_2_ for a predetermined time, the cells were washed twice with PBS. Then 100 μL medium and 10 μL CCK-8 assay kit were added to each well. After 2 h of incubation, the absorbance at 450 nm was measured using a microplate reader (BioTek Instruments, Winooski, VT, USA).

### 4.7. Animal Studies

The animal test was approved by the Institutional Animal Care and Use Committee of Soochow University. Three samples in each bone cement group were inserted into bilateral pockets in the dorsal muscle of female Sprague–Dawley (SD) rats; at the same time, three samples of composite bone cement were inserted into femoral condyle. The implants and surrounding tissue were harvested after four and eight weeks. The samples were imaged with three-dimensional micro-focus computed tomography (micro-CT, Siemens Inveon MM Gantry CT, Munich, Germany), at a voltage of 60 kV and an electric current of 100 μA. Three-dimensional (3D) reconstruction was performed using NRecon software. A cylindrical ROI 4.0 mm in diameter was used for calculating the Mg microspheres’ degradation fraction (cylindrical volume—bone cement volume—undegraded Mg volume/cylindrical volume—bone cement volume), and the means ± standard deviations of the values obtained for three rats in each group were calculated. The harvested samples were also rinsed with distilled water, dried in air, and embedded with resin. The sections of embedded samples perpendicular to bone cement were analyzed by SEM and EDX for morphology and elemental compositions analysis [40].

### 4.8. Statistical Analysis

All statistical analyses were performed using SPSS software. Kruskal–Wallis one-way analysis of variance (ANOVA) tests were followed by Tukey post hoc tests. Unpaired student’s *t*-tests were also used where appropriate. Difference is considered statistically significant when *p* is less than 0.05.

## 5. Conclusions

Partly degradable and bioactive PMMA–Mg composite bone cements have been successfully prepared by incorporating 25%, 41%, or 58% of Mg microspheres into PMMA. This composite bone cement had high compressive strength. Even the weakest strength, 80.30 ± 3.26 MPa for PMMA–25Mg, exceeded the industrial standard (≥70 MPa). In addition, the peak temperature of PMMA–Mg composite bone cement decreased with the content of Mg microspheres. The porous structure of PMMA–Mg composite bone cement, which resulted from the degradation of “magnesium sacrifices,” promoted bony ingrowth in vivo and improved the interfacial bonding strength between bone and bone cement. The PMMA–Mg composite bone cement possessed good biocompatibility and supported cell adhesion and proliferation. Therefore, such a partially degradable PMMA–Mg composite cement may be a promising injectable material for orthopedic surgeries.

## Figures and Tables

**Table 1:** Groups and compositions of cement samples.

Group	Mg Content (wt %)	Liquid Composition (g)		Solid Composition (g)			Mg Microspheres (g)
		MMA	DMPT	PMMA	BPO	BaSO_4_	
PMMA	0	9.4	0.109	17.7	0.3	2	0
PMMA–25Mg	25.3%	9.4	0.109	17.7	0.3	2	10
PMMA–41Mg	40.7%	9.4	0.109	17.7	0.3	2	20
PMMA–58Mg	57.5%	9.4	0.109	17.7	0.3	2	40

## Abbreviations

ANOVA	Analysis of variance
BBC	Bioactive bone cement
BMP-2	Bone morphogenetic protein-2
BPO	Benzoyl peroxide
CaP	Calcium phosphate
CCK-8	Cell counting kit-8
DAPI	4′,6-Diamidino-2-phenylindole
EDX	Energy dispersive X-ray spectroscopy
DMPT	*N*,*N*-Dimethyl-*p*-toluidine
MC	Mineralized collagen
Mg	Magnesium
MG-63	MG-63 cells
MgS	Magnesium sacrifice
MMA	Methyl methacrylate
Micro-CT	Micro-focus computed tomography
PBS	Phosphate-buffered saline
PMMA	Poly (methyl methacrylate)
ROI	Region of interest
SBF	Simulated body fluid
SD	Sprague–Dawley (SD) rat
SEM	Scanning electron microscopy
VEGF	Vascular endothelial growth factor
